# Deep subcutaneous cervical emphysema related to mastoid fracture in an adolescent patient ‐ case report

**DOI:** 10.1002/ccr3.6306

**Published:** 2022-09-12

**Authors:** Dimitrios Paouris, Jana Barkociová, Štefan Pavlík, Irina Šebová

**Affiliations:** ^1^ Clinic of Pediatric Otorhinolaryngology of the Medical faculty of Comenius University and the National Institute of Children's Diseases in Bratislava Bratislava Slovak Republic; ^2^ Department of Radiology of the National Institute of Children's Diseases in Bratislava Bratislava Slovak Republic

**Keywords:** cervical emphysema, hockey puck injury, mastoid fracture, valsalva maneuver

## Abstract

A fracture of the mastoid bone should be considered in the work‐up of a head and neck traumatic injury. A well‐pneumatized mastoid can absorb forceful impacts, protecting middle and inner ear structures. Fractures of the mastoid, followed by Valsalva maneuver can lead to subcutaneous cervical emphysema.

## Graphical abstract

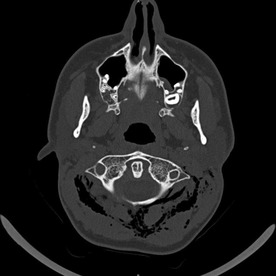

## 1 INTRODUCTION

Subcutaneous emphysema is a condition in which air becomes trapped underneath the dermal layers of skin. The development of subcutaneous air is a benign symptom or an indication of a deeper, more concerning pathologic disease state. A history of head and neck injury can raise suspicion and lead towards the identification of the cause of subcutaneous air collection. Though temporal bone fractures are a commoner occurrence in otorhinolaryngologic traumatology, published cases of traumatic mastoid fractures are extremely rare in English literature.

Air may enter the subcutaneous neck tissues via penetrating or closed non‐penetrating external neck injuries. Air can also be intraluminally dispersed in the neck and chest iatrogenically or due to rupture of a preexisting anatomical abnormality. In addition, cervical and mediastinal emphysema has been reported in a few cases post tonsillectomy. Last but not least, facial trauma and dental or maxillofacial procedures can have similar results.^1^, ^2^

In our report, we present a case of mastoid apex fracture caused by a high‐velocity impact trauma, leading to bilateral subcutaneous cervical emphysema. Though unclear from the anamnesis, we believe the Valsalva maneuver performed by the patient during nose‐blowing caused the spread and entrapment of air through the soft tissue layers of the neck.

## 2 CASE REPORT

A 16‐year‐old adolescent male patient presented to our otorhinolaryngologic emergency service a few hours after being struck by a hockey puck to the left mastoid during a recreational hockey match. The patient denied any discharge from the left ear, signs of dizziness, vertigo, dysphagia, dyspnoea, chest pain, vision problems, or problems with equilibrium. By report, immediately after the hit, he lost his hearing in the left ear for approximately 3–5 min, describing this period as “a feeling of ear fullness”. Subsequently, he describes a subjective restoration of hearing in the left ear, with no tinnitus or earache. The patient has repeatedly blown his nose a few minutes after the injury incident but fails to recall whether it was due to the ear fullness or a feeling of a blocked nose.

Physical examination revealed a 3 × 3.5 cm ecchymosis around the left mastoid tip which was tender on palpation but differed from a Battle's sign. Crepitus was not palpable over the mastoid, while only after thorough palpation was minor crepitation identified posteriorly to the upper third of the sternocleidomastoid muscle ipsilaterally to the injury site. Further palpation revealed minor crepitation cranially at the posterior triangle of the neck contralaterally, with the patient describing a sense of “feeling as if there is something present” upon palpation of the emphysematous cavities – but he described no tenderness. Neck movement was free and painless in all directions. Physical examination did not reveal any focal neurologic findings or deficits, and the facial nerve function was normal (House–Brackmann grade 1). No hematoma or swelling was present on the left external auditory canal, and the left tympanic membrane was intact. There was no fluid collection present in the middle ear. Rinne test was positive bilaterally, Weber test showed no lateralization, while tympanometry was of A‐type, with stapedial reflex being present ipsilaterally at a signal of 85 dB/500 Hz bilaterally. For suspicion of subcutaneous emphysema and a temporal bone fracture, a High‐Resolution Computed Tomography (HRCT) scan was performed.

**FIGURE 1:**
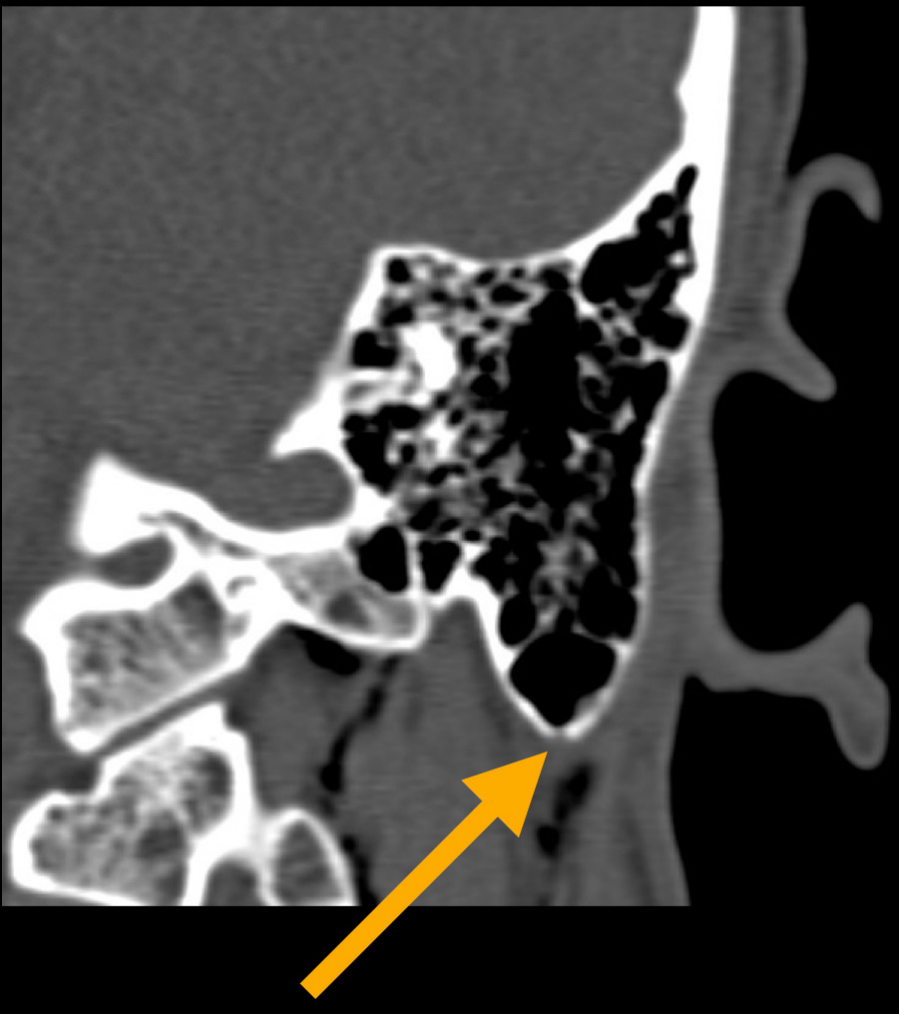
Coronal plane HRCT scan – Left side: caudal mastoid apex fracture (yellow arrow), with presence of SCE

**FIGURE 2:**
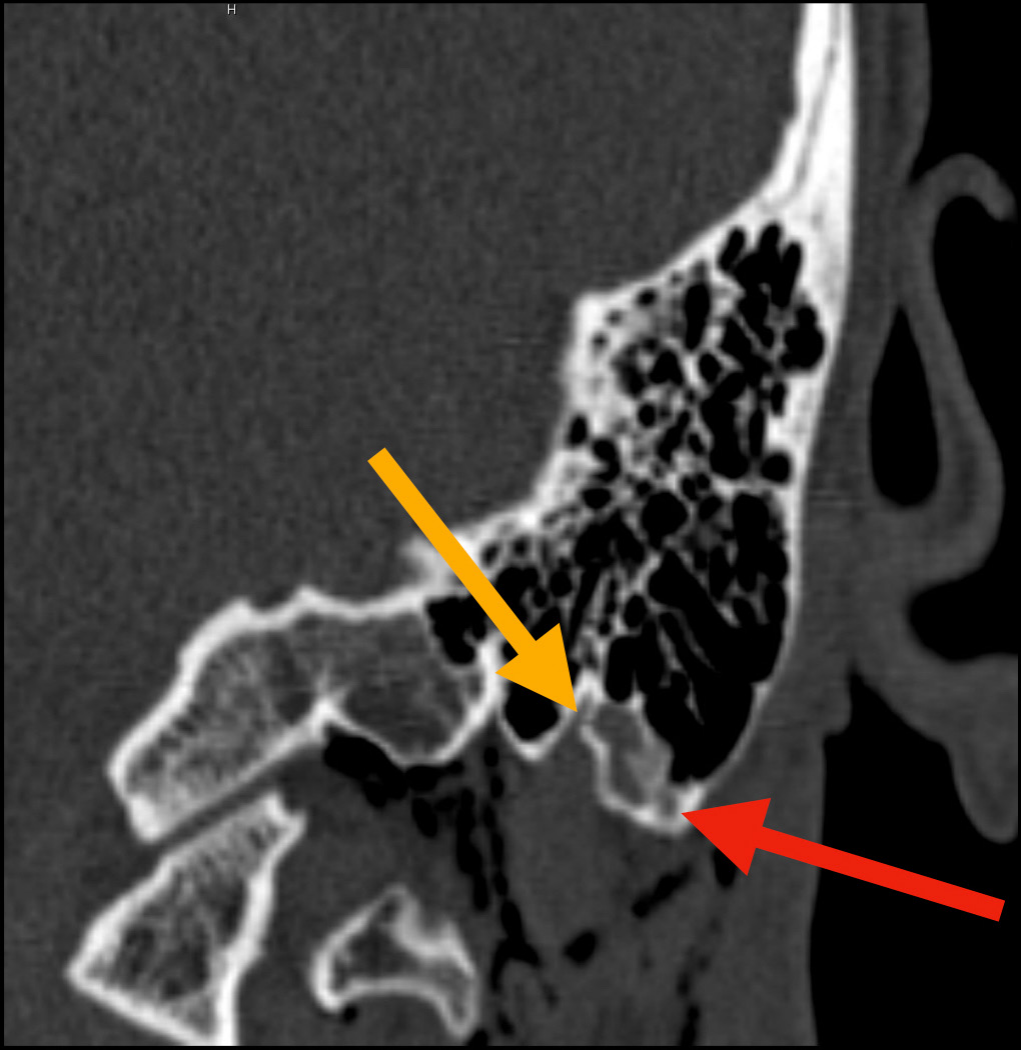
Coronal plane HRCT scan – Left side: medial mastoid apex fracture (yellow arrow), fluid within the mastoid air cells (red arrow), presence SCE at the level of C1, lateral to the occipital condyle

The HRCT scan of the head and neck demonstrated no intracranial injury. No mass effect or midline shift on the brain was observed. No temporal bone fracture was present, but two isolated linear fractures on and around the mastoid apex with a compromise of the pneumatization of the surrounding air cells were detected on the left side. Extensive left cervical emphysema extending from the fracture to the skull base ipsilaterally and the cervical soft tissues bilaterally, along with the adjacent musculature, was identified (Figures 1, 2). There was no involvement of the lower neck below the level of the thyroid cartilage. There was no violation of the otic capsule, and ossicular alignment was normal.

**FIGURE 3:**
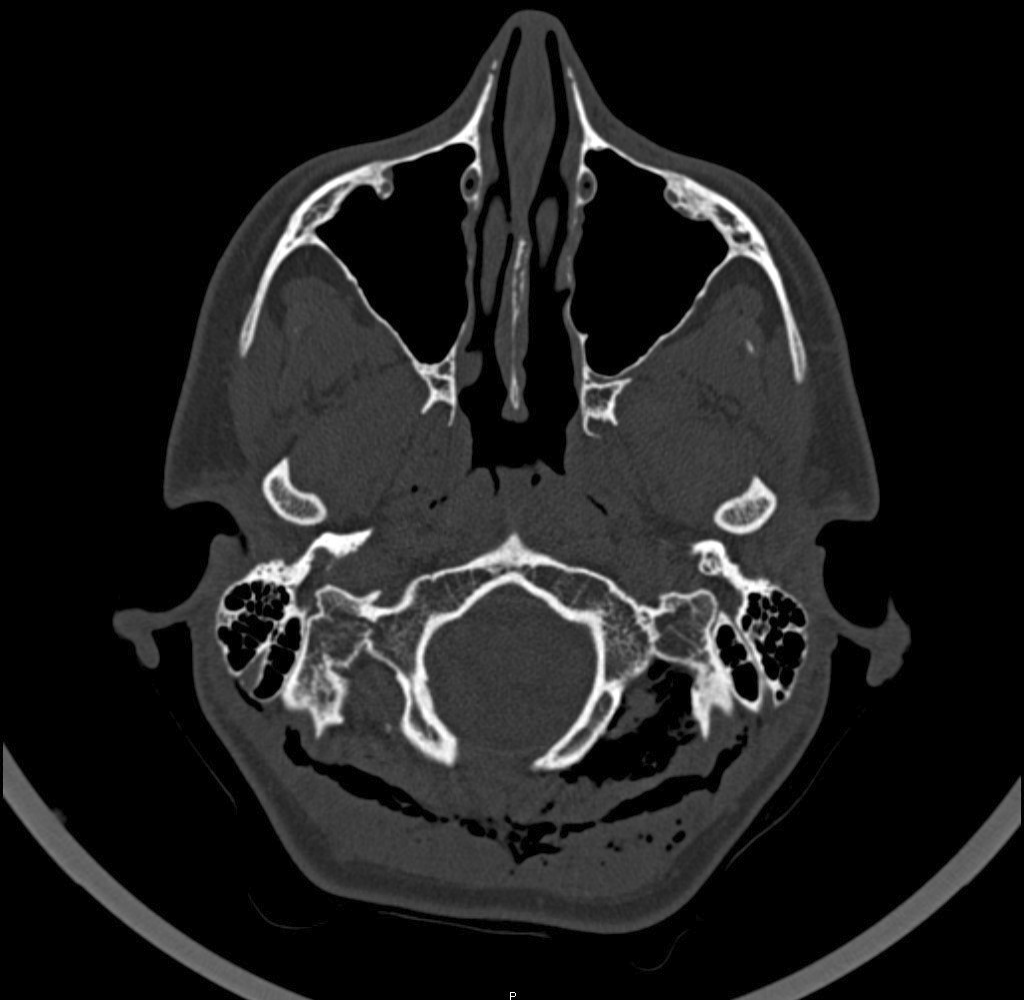
Axial plane HRCT scan – bilateral SCE evident at the level of C1, between the suboccipital muscles bilateraly

**FIGURE 4:**
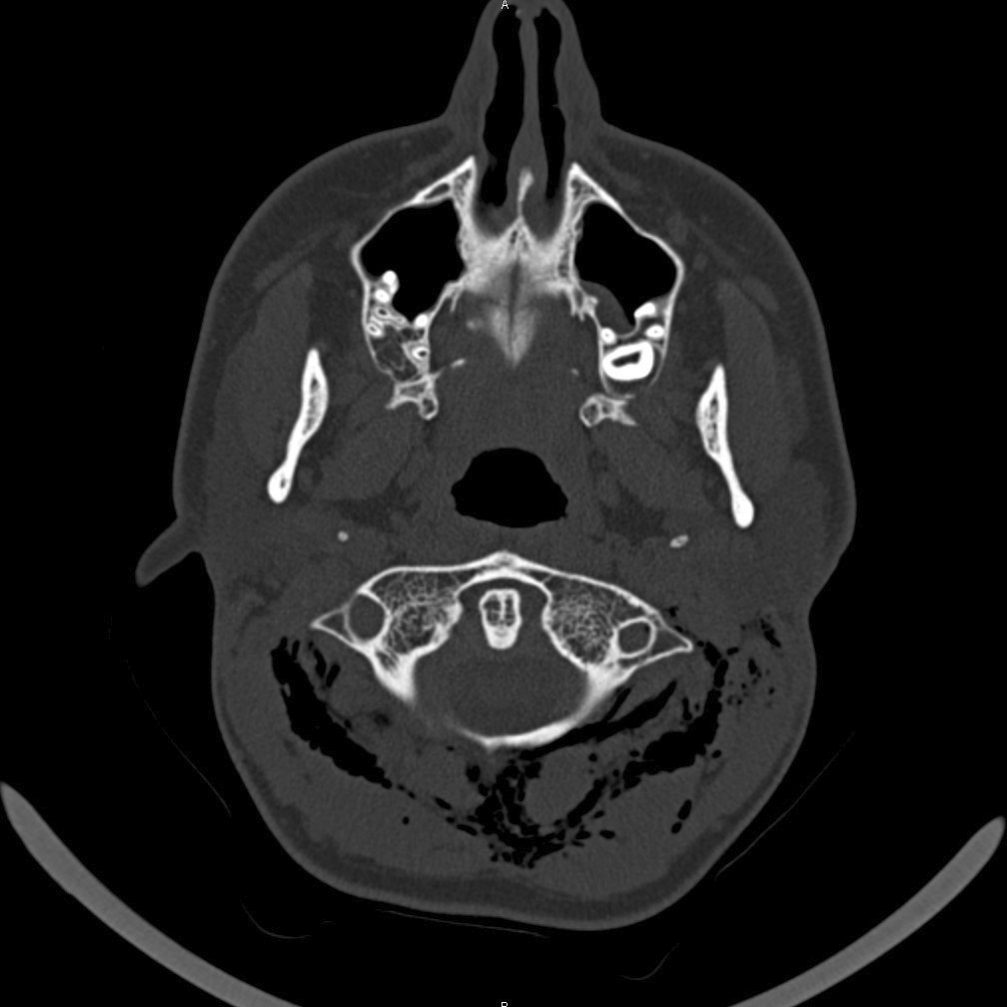
Axial plane HRCT scan – bilateral SCE evident at the level of C1‐C2, between the suboccipital muscles bilateraly

The neck CT scan revealed extensive bilateral subcutaneous and deep cervical emphysema extending from the posterior occiput caudally. Air is visible in the region laterally to the occipital condyle at the levels of C1–C2 and the space between the suboccipital muscles bilaterally (Figures 3, 4).

The chest X‐ray image demonstrated no mediastinal extension of the cervical emphysema.

The patient was admitted for monitoring while intravenous analgesia and antibiotic treatment (amoxicillin/clavulanate) were administered. The hospitalization lasted for 3 days and was uneventful. The patient had no signs of respiratory distress and did not complain of pain. Moreover, he was restricted to bed and strongly advised to refrain from coughing or nose blowing. Seventy‐two hours after the accident, an ultrasound of the neck revealed a hardly detectable amount of air, while the control chest x‐ray was again without any pathological findings. At the outpatient otorhinolaryngologic follow‐up after 10 days, he was asymptomatic, demonstrating no oto/audiological or other sequelae from the injury, with his audiogram being normal.

## 3 DISCUSSION

Subcutaneous cervical emphysema (CSE) due to mastoid fracture is a rare occurrence, with only four reported cases in the English literature to date.^1^, ^3^, ^4^, ^5^ The most common pathologies connected with mastoid fractures are CSF leakage and pneumocephalus.^6^ In our case study, the injury was caused by blunt force trauma by a hockey puck. A standard hockey puck can weigh around 0.16 kg and travel with a speed of around/more than 80 km/h, causing a very high impact force injury. Two of the reported cases include exertion of physical violence with direct hits to the mastoid area being suffered by the patient,^4^, ^5^ while one case was caused after some accidental sharp object penetration injury.^3^ Various reported cases in the published literature regarding head and neck subcutaneous emphysema (SE) formation as a complication of athletic trauma are found. The impact of play objects/balls (e.g., baseball, tennis ball, hockey puck), which can travel with high velocity, leads to the transfer of high amounts of energy to the impacted area, resulting in trauma and fractures.^1^, ^3^ There are also reports of SE following fall or dive‐related injuries.^7^, ^8^ Due to its air cell honeycomb‐like pattern, it has been hypothesized that a function of the mastoid air cell system (MACS) is to act as a damping barrier, protecting the middle ear and its contents, the brain, and the otic capsule. Also, the higher the pneumatization, the better the protective effects the mastoid air cell system (MACS) can provide.^9^ The degree of pneumatization in our case was extensive. We believe this case study is another proof of the protective cushioning the mastoid bone provides, similar to the one provided by the paranasal sinuses.

This present case features a high‐velocity injury with focal impact to the mastoid bone that resulted in a minor linear fracture. As far as mastoid impact fractures are concerned, the impact can displace mastoid air into the soft tissues. Therefore, the amount of exerted force (e.g., severe high‐velocity impact), and the extent of mastoid fracture (e.g., large comminuted fracture) can impact the amount of air leaking out of the mastoid, as seen in the published literature.^1^, ^3^, ^4^, ^5^, ^10^

The extent of the injury in our case, though, does not fully correspond to the amount of air dispersed throughout the cervical soft tissue structures. We believe that when the patient blew his nose and performed the Valsalva maneuver, he caused air propagation. By performing the Valsalva maneuver, air is forced through the Eustachian tube, past the non‐compressible middle ear, finally reaching the MACS with the existing air content of the mastoid cells transiently pressurized. In the case of a mastoid fracture, a decompression valve is formed, and air can be squeezed out and dissected through the fascial planes and the attached musculature.^11^, ^12^

Our patient did not have any injury to the temporal bone or otic capsule nor did he present with any clinical detectable facial weakness. We believe that the transient hearing loss resulted from the pressure difference created following air mobilization from the mastoid to the middle ear. Audiological measurement has shown normal hearing, also without any other sequelae like tinnitus.

Careful physical examination is of paramount importance when encountering a patient with trauma of the facial skeleton or the temporal bone. Crepitus can be revealed many times only after careful palpation of the soft tissues, while pain and limitation of movement can be present, but not in every case. A thin slice/high‐resolution CT scan is recommended as a careful evaluation of the temporal bone is crucial. In addition, temporal bone fractures most probably will present with indirect findings like the presence of emphysema in the surrounding area. Evaluation of other facial structures on the CT scan is also vital since fractures of the facial skeleton may cause cervical emphysema.^13^

Management of SCE varies according to the cause and associated conditions. In cases of open facial fractures, reduction, fixation, and antibiotics may be indicated. A consultation by the neurosurgical service is always an option. Prophylactic antibiotics are advisable in complicated mastoid fractures, while there is a controversy of opinions regarding uncomplicated ones. The SCE cavity may be filled with fluid and get inflamed, but the course of most SCE is to resolve spontaneously. The patients should be strictly advised to keep their mouth open during sneezing and coughing as well as to avoid wind instruments, nose‐blowing, and air traveling for a few weeks.^2^, ^5^

## 4 CONCLUSION

The present case study describes a high‐velocity mastoid bone injury causing SCE. A fracture of the mastoid bone should be included in the work‐up of a head and neck traumatic injury. A well‐pneumatized mastoid can absorb forceful impacts, protecting middle and inner ear structures. On the set of a mastoid fracture, the Valsalva maneuver can have a significant role in SCE formation but is not a prerequisite since an adequate force of impact can by itself disseminates air from the mastoid to the surrounding soft tissues. Patients may initially present with minimal symptoms, but their condition may deteriorate rapidly or insidiously, especially in settings of mediastinal emphysema.

## AUTHOR CONTRIBUTIONS

Dimitrios Paouris: contributed to writing—original draft. Jana Barkociová: contributed to project administration, writing—review and visualization. Štefan Pavlík: contributed to review and visualization. Irina Šebová: contributed to supervision and review.

## CONFLICT OF INTEREST

None declared.

## CONSENT

Written informed consent was obtained from the patient to publish this report in accordance with the journal's patient consent policy.

## Data Availability

Data sharing not applicable to this article as no datasets were generated or analysed during the current study
